# Treatment with *Terminalia chebula* Extract Reduces Insulin Resistance, Hyperglycemia and Improves SIRT1 Expression in Type 2 Diabetic Rats

**DOI:** 10.3390/life13051168

**Published:** 2023-05-11

**Authors:** Ojaskumar D. Agrawal, Yogesh A. Kulkarni

**Affiliations:** 1 Shobhaben Pratapbhai Patel School of Pharmacy & Technology Management, SVKM’s NMIMS, V.L. Mehta Road, Vile Parle (W), Mumbai 400056, India; 2 Vivekanand Education Society’s College of Pharmacy, University of Mumbai, Chembur (E), Mumbai 400074, India

**Keywords:** *Terminalia chebula*, insulin resistance, tannins, type 2 diabetes, hyperglycemia, high-fat diet

## Abstract

Background: *Terminalia chebula* Retz., Family Combretaceae (*T. chebula*) is one of the important plants mentioned in Ayurveda, a traditional system of medicine. The present work was designed to study the effect of the aqueous extract of *T. chebula* fruits in type 2 diabetic rats. Methods: The aqueous extract of the fruits was prepared by the double maceration technique. The extract was subjected to HPTLC analysis, which showed the presence of ellagic acid and gallic acid. Type 2 diabetes was induced in rats with a low dose of Streptozotocin (35 mg/kg) after administering a high-fat diet for fourteen days. Diabetic animals were treated with 500 and 1000 mg/kg of aqueous extract of *T. chebula* fruits for six weeks. Results: Diabetic rats showed a significantly (511.7 ± 17.6) (*p* < 0.001) high plasma glucose level compared to the normal group (106 ± 3.358). The *T. chebula* treatment group showed a significant (*p* < 0.001) reduction in plasma glucose at 500 mg/kg (394.3 ± 10.35) and 1000 mg/kg (368.6 ± 30.08) doses when compared with the diabetic control group. Treatment with aqueous extract significantly reduced lipid parameters in diabetic animals when compared to the animals in the diabetic control group. Treatment with extract at a dose of 500 mg/kg and 1000 mg/kg showed a significant reduction in AST (*p* < 0.01, *p* < 0.001) when compared with diabetic control rats. Treatment with extract significantly reduced ALT at 500 mg/kg (*p* < 0.05) and 1000 mg/kg (*p* < 0.001) doses when compared with diabetic control rats. The extract treatment improved insulin sensitivity and insulin sensitivity index (ISI) and significantly decreased HOMR-IR. Treatment with *T. chebula* aqueous extract at 1000 mg/kg significantly increased the level of GSH (*p* < 0.05) when compared to diabetic control rats. *T. chebula* treatment at 1000 mg/kg significantly increased levels of CAT (*p* < 0.01). Histopathology of pancreatic tissue revealed that the extract has a protective effect against the damage caused by hyperglycemia. Immunohistochemistry of pancreatic tissue showed increased expression of SIRT1 in diabetic animals treated with the extract. Conclusions: The results of the present study indicate that the extract of *T. chebula* has significant effects in the management of type 2 diabetes.

## 1. Introduction

Diabetes mellitus (DM) is one of the most prevalent diseases, in which the body either does not produce enough insulin or resists its action. Symptoms of diabetes include increased thirst, frequent urination, hunger, fatigue, and blurred vision [1]. Treatment options include diet, exercise, medication, and insulin therapy. Compared to type 1 diabetes, the number of patients with type 2 diabetes is greater [2]. It is estimated that, in 2030, 643 million people will have diabetes, which is projected to reach 783 million by 2045 [3]. In type 2 diabetes, hyperglycemia is the result of inadequate production of insulin and inability of the body to respond fully to insulin which is called as insulin resistance. In insulin resistance, insulin is ineffective and, therefore, initially prompts an increase in insulin production to reduce rising glucose levels, but over time a state of relatively inadequate production of insulin can develop [4]. In some patients with type 2 diabetes, the condition becomes more critical, and the pancreas will be exhausted completely, which will further produce lower amounts of insulin, causing higher blood sugar levels [3].

The development of type 2 DM includes several lifestyle-related factors among them are a sedentary lifestyle, alcohol consumption, lack of physical activity, and cigarette smoking [5]. As a result of these, dyslipidemia is one of the most commonly observed condition in patients with diabetes [6,7]. It is the imbalance in the body’s lipids, such as triglycerides, cholesterol, low-density lipoprotein, and high-density lipoprotein. This condition is linked to high-fat diet and exposure to tobacco, or it may be genetic in nature as well. It can results in heart-related diseases with moderate to severe complications [6,8]. Cardiovascular diseases is the foremost concern for mortality and morbidity in T1DM and T2DM in both men and women [9].

Current diabetes treatment includes Sulfonylureas, Thiazolidinediones, Glinides, GLP-1 receptor agonists, DPP-4 inhibitors, insulin therapy, and SGLT2 inhibitors [10]. Sulfonylureas produce low blood sugar, upset stomach, skin rash or itching, and weight gain; biguanides/metformin can lead to kidney complications, upset stomach, tiredness or dizziness, and metal taste alpha-glucosidase inhibitors produce gas, bloating, and diarrhea [11]. So, there is an unmet need to discover new treatment options for management of diabetes.

Several medicinal plants have been explored for antidiabetic and related beneficial effects in the treatment of diabetes, namely *Allium sativum* [12,13], *Eugenia jambolana* [14], *Momordica charantia* [15], *Ocimum sanctum* [16,17], *Phyllanthus amarus* [18,19,20], *Pterocarpus marsupium* [21], *Tinospora cordifolia* [22], *Trigonella foenum graecum* [23], and *Withania somnifera* [24]. *Terminalia chebula* Retz. (*T. chebula*) is one of the crucial plants mentioned in Ayurveda, a traditional system of medicine. It shows presence of ellagic acid, gallic acid, ethyl gallate, chebulinic acid, tannic acid, chebulin, punicalagin, arjunglucoside I, luteolin, terflavin A, etc. [25]. Phytochemicals and extracts of *T. chebula* have been reported for various pharmacological effects, such as antidiabetic activity [26,27,28,29] and hepatoprotective effects [30,31]. Several chemical compounds, such as tannins, polyphenols, and triterpenoids, were isolated from *T. chebula* [32]. It is effective in the inhibition of cutaneous photodamage [33] and has antioxidant effects [34,35,36,37,38,39]. It has also been reported to have wound-healing properties [40,41]. Authors have also reported free radical scavenging activity of *T. chebula* [38,42]. Karla and co-researchers have performed chemical characterization of the extract by HPLC and found the presence of phytoconstituents such as gallic acid, ellagic acid, gallic acid, corilagin, chebulic, chebulinic acid, and chebulagic acid [43]. In addition to the pharmacological activity, phytoconstituent analysis is also performed by several researchers, which includes High-performance capillary electrophoresis (HPEC) analysis of chebula fruits by Ding and co-researchers. They reported presence of hydrolyzable tannins such as chebulagic acid and chebulinic acid [44]. Juang and coresearchers have reported presence of gallic and ellagic acid along with other active phytoconstituents [45]. Kundu and coresearchers have reported presence of flavanol glycosides, triterpenoids, coumarin conjugated, and phenolic compounds in *T. chebula* [46]. Considering the presence of the active phytoconstituents and based on the literature search, it was observed that there are no systematic investigations on *T. chebula* for its possible effects on type 2 diabetes mellitus. Therefore, the present work was designed to study the effects of an aqueous extract of *T. chebula* in animal models of type 2 diabetes.

## 2. Materials and Methods

### 2.1. Plant Material

The dried fruits of *T. chebula* were purchased from the D.G. Ayurvedic Sangrah, Mumbai, India, in November 2018. The plant material was authenticated by Dr. (Mrs.) A.S. Upadhye, Scientist, Herbal Drug Administration at Agharkar Research Institute, Pune, Maharashtra, India. A voucher specimen (Auth. 18–205) was deposited in the Department for future use.

### 2.2. Chemicals

Yeast extract and DL-methionine were purchased from Molychem, India. Cholesterol was obtained from Loba Chemie Pvt. Ltd., Maharashtra, India. Casein was procured from Clarion casein limited, India. The lard was purchased from the local vendor. Streptozotocin was ordered from Sigma Aldrich (St. Louis, MO, USA), and sodium chloride was purchased from SD Fine Chemicals, India. From Transasia Biomedicals Ltd., India, diagnostic kits were obtained. Glipizide tablets was purchased from the local market. Ellagic acid and gallic acid were procured from MP Biomedicals, USA and Sigma-Aldrich, USA respectively.

### 2.3. Preparation of Aqueous Extract

Kernels of dried fruits of *T. chebula* were removed, powdered, and used for the preparation of aqueous extract by using the double maceration technique. Powdered fruits (500 g) were macerated with 5000 mL of distilled water for seven days. At the end of the seventh day, the extract was filtered through muslin cloth, followed by filter paper and the filtrate was stored in the refrigerator till further use. The marc was again kept for maceration with fresh distilled water for seven days. The extract was filtered through muslin cloth, followed by filter cloth after seven days. The filtrate obtained in this step was mixed with the filtrate obtained in the first step. The extract was concentrated on a water bath (Labline, India) and was stored in a refrigerator until further use [47]. The percentage yield of semisolid extract was found to be around 34% *w*/*w*.

### 2.4. Quantification of Ellagic Acid and Gallic Acid in the Aqueous Extract by HPTLC Method

Quantification of ellagic acid and gallic acid from the aqueous extract of *T. chebula* was performed by the High-Performance Thin Layer Chromatography (HPTLC) method. HPTLC analysis was carried out by using CAMAG HPTLC (Switzerland) equipped with visionCATS HPTLC Software, CAMAG Linomat V applicator, CAMAG Hamilton Micro syringe (100 µL), CAMAG TLC Scanner 4 (4.6 mm × 25 cm, 5 μm), CAMAG UV Cabinet (254 and 366 nm), and CAMAG Twin Trough Chamber (20 × 10 cm). The separation was carried out using a solvent system of Methanol: Water: Formic acid (4:6:0.5) *v*/*v*/*v,* and the stationary phase was pre-coated alumina backed, HPTLC Plate Silica gel 60 RP-18 (Merck). The scanning of the developed plate was performed at 254 nm. Ellagic acid and gallic acid reference material were used as standards for quantifying tannins in the extract. The standard solutions of ellagic acid and gallic acid of the concentration of 100 μg/mL were prepared in methanol and used for HPTLC analysis.

### 2.5. Experimental Animals

Male *Sprague Dawley* rats (160–170 g) were purchased from the National Institute of Biosciences (Pune, India). The animals were housed in the animal facility at a temperature and relative humidity of 22 ± 2 °C and 75 ± 5%, respectively. The experimental animals were fed a basal regular pellet diet (Nutrimix Laboratory Animal Feed, MS, India) throughout the experiment. The high-fat diet consisting of 25% protein, 58% fat, and 17% carbohydrate was prepared in lab [48]. The high-fat diet composition per kilogram includes normal pellet diet 365 g, pork lard 310 g, Casein 250 g, Cholesterol 10 g, Vitamin D mineral mix 60 g, DL-Methionine 3 g, yeast powder 1 g, Sodium Chloride 1 g.

### 2.6. Induction of Diabetes Mellitus

Type 2 diabetes was induced using a low dose of Streptozotocin (STZ) (35 mg/kg, *i.p.*, using 0.1 M cold citrate buffer at pH 4.4, volume based upon body weight) after two weeks of dietary modification using a high-fat diet [48]. The experimental animals with blood glucose levels above 250 mg/dL were selected for the research study [49].

Group I: Normal control (received purified water).

Group II: Diabetic control rats (received purified water).

Group III: Diabetic rats were administered with *T. chebula* fruits aqueous extract (500 mg/kg body weight/day/rat) in distilled water.

Group IV: Diabetic rats were administered with *T. chebula* fruits aqueous extract (1000 mg/kg body weight/day/rat) in distilled water.

Group V: Diabetic rats were administered with Glipizide (standard drug) (5 mg/kg body weight/day/rat) in 0.5% Sodium Carboxy Methyl Cellulose (Na CMC).

The treatment was given for six weeks by oral route with the help of oral gavage. High-fat diet was given during the period of treatment.

### 2.7. Pancreatic Tissue Collection and Processing

At the end of experiment, animals from different treatment groups were humanely sacrificed, and the pancreas was isolated to determine oxidative stress parameters, histopathological studies, and immunohistochemistry.

### 2.8. Evaluation of Parameters

#### 2.8.1. Body Weight

The body weight of the animals from different groups was recorded at the end of the study.

#### 2.8.2. Estimation of Plasma Glucose and Lipids

Blood samples of animals were collected, and plasma was separated. Plasma glucose level and lipid markers such as total cholesterol, triglyceride, high-density lipoprotein, and low-density lipoprotein were analyzed by kits procured from Transasia Bio-Medicals Ltd., India, and evaluation was performed using a Biochemical Analyzer, (Erba Chem-7, Germany) [49].

#### 2.8.3. Estimation of Liver Enzymes

Alanine Transaminase (ALT) and Aspartate Aminotransferase (AST) were measured as per the protocol provided by Transasia Bio-Medicals Ltd., India.

#### 2.8.4. Estimation of Glycohemoglobin Content

Using the resin (ion exchange) method, as per the procedure mentioned in the kit supplied by Transasia Bio-Medicals Ltd., India, Glycohemoglobin content was determined [49].

#### 2.8.5. Estimation of Glucose Tolerance and Insulin Resistance

##### Estimation of Oral Glucose Tolerance Test (OGTT)

An OGTT was performed as per the previously described method [49].

##### Measurement of Plasma Insulin (PI), Insulin Sensitivity Index (ISI), and Homeostatic Model Assessment-Insulin Resistance (HOMA-IR)

Insulin level was estimated in plasma samples using rat insulin enzyme-linked immunosorbent assay (ELISA) kits supplied by Abbkine, USA.

Homeostatic Model Assessment-Insulin Resistance (HOMA-IR) and insulin sensitivity index (ISI) were calculated using the following formula:

ISI = ln (1/FINS × FBG)(1)

HOMA-IR = (Glucose × Insulin)/22.5(2)

where the concentration of insulin is expressed in mIU/L and glucose in mmol/L.

#### 2.8.6. Pancreatic Tissue Collection and Processing

##### Determination of Oxidative Stress Parameters

Tissue homogenate (10% *w*/*v*) was prepared in phosphate buffer 50 mm (pH 7.4). Oxidative stress parameters such as Malondialdehyde, Superoxide Dismutase, Catalase, and Reduced Glutathione were estimated in pancreatic tissue homogenate. MDA and GSH levels were also estimated in pancreatic tissue homogenate. CAT was evaluated in the post-nuclear homogenate fraction, whereas SOD was estimated in the post-mitochondrial fraction of homogenate [50,51,52,53].

##### Histopathology of Pancreatic Tissue

This study was processed as reported earlier [49,54]. The stained slides were studied under a photographic microscope (Motic, Canada) for histopathological changes.

##### Immunohistochemistry of Pancreatic Tissue

As reported earlier, the pancreatic tissue immunohistochemical staining was carried out to study expression of SIRT1 [54].

### 2.9. Statistical Analysis

The data analysis was performed using software, GraphPad Prism for Windows ver. 5.00. The data are expressed in Mean ± SEM. Significant differences between the experimental animal groups were assessed by one-way analysis of variance test (ANOVA) followed by Bonferroni’s multiple comparison tests to determine the significance level. *p*-value less than 0.05 was considered significant.

## 3. Results

### 3.1. Identification of Ellagic Acid and Gallic Acid in Aqueous Extract of Fruits of T. chebula by HPTLC Method

**Figure 1:**
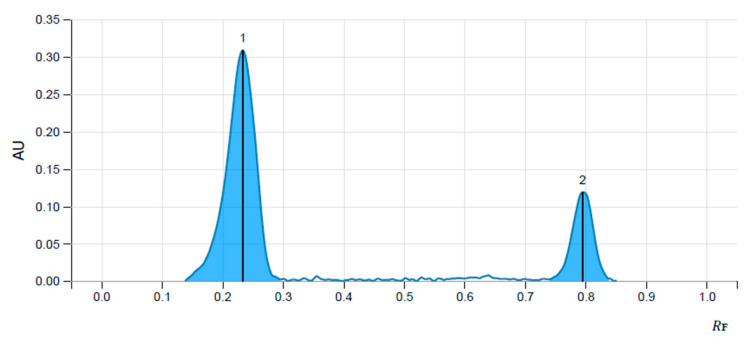
Chromatogram of Standard ellagic acid (1) and gallic acid (2).

**Figure 2:**
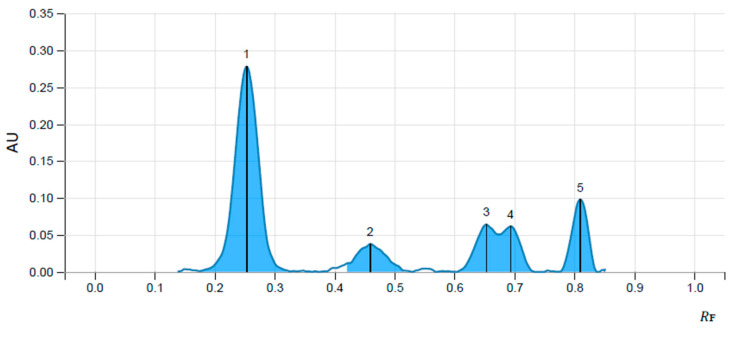
Chromatogram of aqueous extract of fruits of *T. chebula* showing the presence of ellagic acid (1) and gallic acid (5).

Using the mobile phase mentioned in the materials and methods section, reproducible and sharp ellagic and gallic acid peaks with symmetry as per the limit, were obtained. Hence, it was considered as an optimized condition for standardizing extraxt for ellagic and gallic acid. The retention factor for standard ellagic acid and gallic acid was found to be 0.234 and 0.795, respectively, whereas the peaks of ellagic acid and gallic acid in the extract were obtained at a similar retention factor as that of standards, confirming the presence of the same in the extract. The percent content of ellagic acid and gallic acid in the *T. chebula* aqueous extract was found to be 0.0018% and 0.0077%, respectively. Chromatograms of standards and extract are shown in Figure 1 and Figure 2, and the chromatographic data are presented in Table 1.

### 3.2. Body Weight

**Figure 3:**
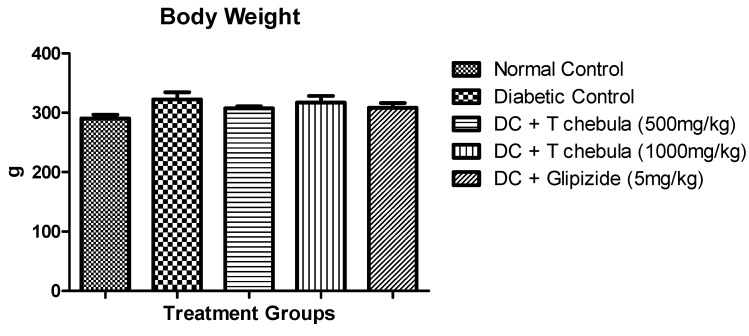
Effect of *T. chebula* extract on body weight. Values are expressed as Mean ± SEM (*n* = 6). (DC: diabetic control; *T chebula*: *Terminalia chebula*).

The effect of *T. chebula* on the body weight of animals in different treatment groups is presented below (Figure 3) The animals in diabetic control group did not show any significant changes in body weight when compared with normal control group. The animals in treatment group did not show any significant changes in body weight when compared to diabetic control group.

### 3.3. Effect of Terminalia chebula Extract on Plasma Glucose and Lipid Parameters in Diabetic Rats

#### 3.3.1. Plasma Glucose

**Figure 4:**
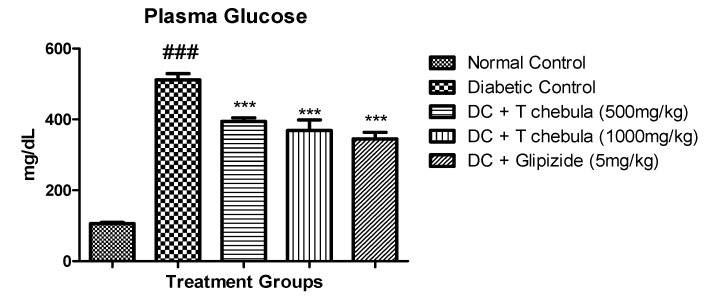
Effect of *T. chebula* extract on plasma glucose. Values are expressed as Mean ± SEM (*n* = 6). *** *p* < 0.001 when treatment groups were compared with diabetic control. ### *p* < 0.001 when the diabetic control group was compared to the normal control group. (DC: diabetic control; *T chebula*: *Terminalia chebula*).

Diabetic rats showed a significantly (*p* < 0.001) high plasma glucose level (511.7 ± 17.6) compared to the normal group (106 ± 3.358). The *T. chebula* treatment group showed a significant (*p* < 0.001) reduction in plasma glucose at 500 mg/kg (394.3 ± 10.35) and 1000 mg/kg (368.6 ± 30.08) doses when compared with the diabetic control group. The results are shown in Figure 4.

#### 3.3.2. Lipid Parameters

**Figure 5:**
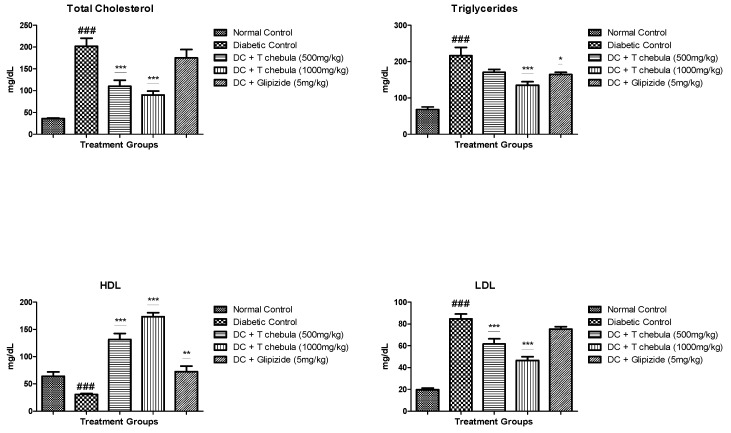
Effect of *T. chebula* treatment on plasma lipids in type 2 diabetic rats. Values are expressed as Mean ± SEM (*n* = 6). * *p* < 0.05, ** *p* < 0.01, and *** *p* < 0.001 when treatment groups were compared with diabetic control. ### *p* < 0.001 when the diabetic control group was compared to the normal control group. (DC: diabetic control; *T chebula*: *Terminalia chebula*).

The effect of *T. chebula* on plasma levels of triglyceride, total cholesterol, LDL, and HDL is shown in Figure 5. The diabetic control group showed a significant (*p* < 0.001) increase in the plasma TC (201.9 ± 18.48), TG (216.1 ± 23.08), and LDL (84.6 ± 4.513) levels compared with the normal control group. The plasma HDL (30.69 ± 1.731) level was also significantly lowered in a diabetic control animals (*p* < 0.001) than in normal animals (64.15 ± 7.721). Treatment with aqueous extract at 1000 mg/kg significantly (*p* < 0.001) reduced triglyceride levels (135.1 ± 9.668) in diabetic animals when compared with the diabetic control group (216.1 ± 23.08). Total cholesterol level was also markedly reduced in diabetic animals (*p* < 0.001) after treatment with *T. chebula* aqueous extract at 500 mg/kg (110.4 ± 13.41) and 1000 mg/kg (90.17 ± 8.738) doses when compared with diabetic control rats. *T. chebula* aqueous extract at both doses showed a significant increase in HDL level (*p* < 0.001) in diabetic rats. Treatment with *T. chebula* aqueous extract showed a significant reduction (*p* < 0.001) in LDL levels at both doses (*p* < 0.001).

### 3.4. Effect of T. chebula Extract on Liver Enzymes

**Figure 6:**
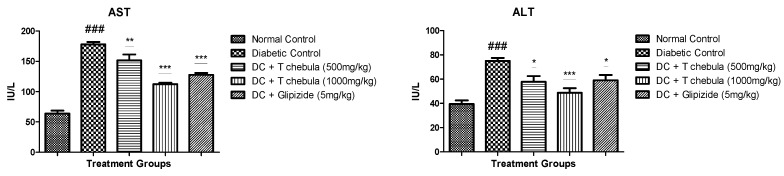
Effect of *T. chebula* on Liver Enzymes. All values are expressed as Mean ± SEM (*n* = 6). * *p* < 0.05, ** *p* < 0.01, and *** *p* < 0.001 when treatment groups were compared with diabetic control. ### *p* < 0.001 when the diabetic control group was compared with the normal control group. (DC: diabetic control; *T chebula*: *Terminalia chebula*).

Diabetic animals showed a significant (*p* < 0.001) increase in levels of AST (178 ± 4.123) and ALT (75.04 ± 2.435) compared to normal rats. Treatment with *T. chebula* aqueous extract at 500 mg/kg (151.6 ± 9.888) (*p* < 0.01) and 1000 mg/kg (112.3 ± 2.36) (*p* < 0.001) dose showed a significant reduction in AST when compared with diabetic control rats. Treatment with *T. chebula* aqueous extract significantly reduced ALT at 500 mg/kg (151.6 ± 9.888) (*p* < 0.05) and 1000 mg/kg (112.3 ± 2.36) (*p* < 0.001) dose when compared with diabetic control rats. Standard Glipizide treatment significantly (59.04 ± 4.463) (*p* < 0.05) reduced the level of ALT in comparison to diabetic control rats (Figure 6).

### 3.5. Effect of T. chebula on Glycohemoglobin

**Figure 7:**
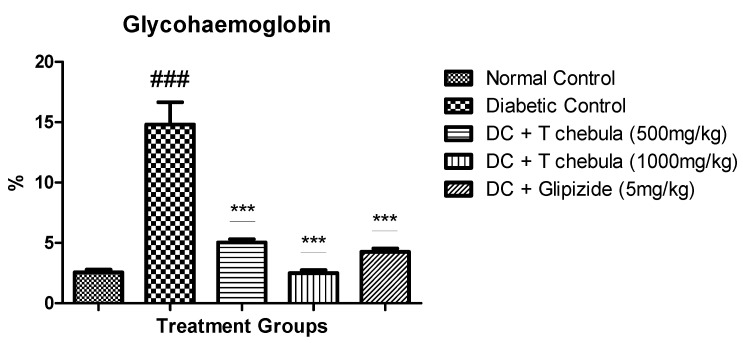
Effect of *T. chebula* treatment on HbA1c (%Hb) in type 2 diabetic rats. Values are expressed as Mean ± SEM (*n* = 6), *** *p* < 0.001 when the treatment group is compared with the diabetic control group. ### *p* < 0.001 when the diabetic control group is compared to the normal control. (DC: diabetic control; *T chebula*: *Terminalia chebula*).

Diabetic animals showed a significant increase (14.82 ± 1.845) (*p* < 0.001) in glycohemoglobin levels in the blood when compared with the normal group (2.554 ± 0.2412), while treatment with *T. chebula* aqueous extract at 500 mg/kg (5.034 ± 0.269) and at 1000 mg/kg (2.499 ± 0.242) doses showed a significant reduction (*p* < 0.001) in glycohemoglobin level when compared with the diabetic control group (Figure 7).

### 3.6. Glucose Tolerance and Insulin Resistance Measurement

#### 3.6.1. Effect of *T. chebula* on Oral Glucose Tolerance Test (OGTT)

**Figure 8:**
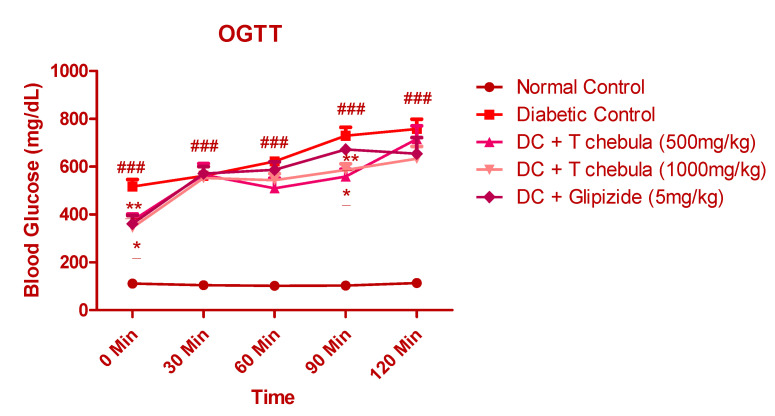
Effect of *T. chebula* aqueous extract on OGTT. Values are expressed as Mean ± SEM (*n* = 6). * *p* < 0.05, ** *p* < 0.01 when treatment groups were compared with diabetic control. ### *p* < 0.001 when the diabetic control group was compared with the normal control groups. (DC: diabetic control; *T chebula*: *Terminalia chebula*).

Figure 8 illustrates the results of the oral glucose tolerance test performed at the end of the study in 8 h fasted rats. The results showed that the plasma glucose level at 0, 30, 60, 90, and 120 min remained significantly higher in the diabetic control group when compared with the normal control group (*p* < 0.001) and other treatment groups (*p* < 0.05 and 0.01). 

#### 3.6.2. Measurement of Plasma Insulin (PI), Insulin Sensitivity Index (ISI), and Homeostatic Model Assessment-Insulin Resistance (HOMA-IR)

**Figure 9:**
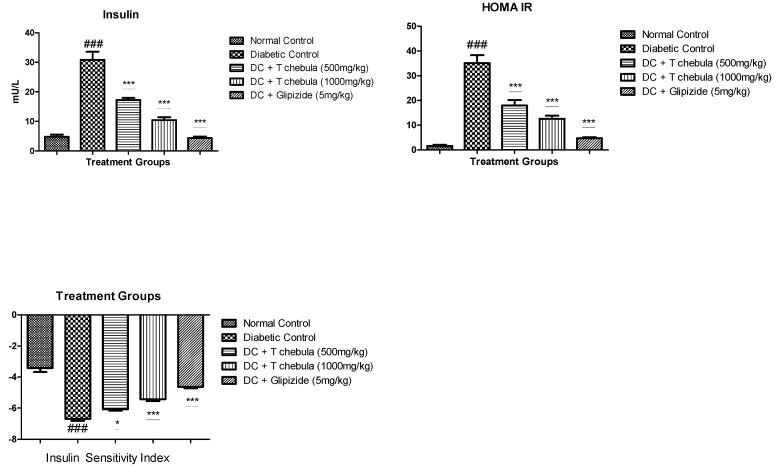
The *T. chebula* aqueous extract treatment effect on A. Plasma Insulin, B. HOMA-IR, and C. Insulin sensitivity index (ISI) in type 2 diabetic rats. Values are expressed as Mean ± SEM (*n* = 6). * *p* < 0.05 and *** *p* < 0.001 when treatment groups were compared with diabetic control. ### *p* < 0.001 when the diabetic control group compared to the normal control group. (DC: diabetic control; *T chebula*: *Terminalia chebula*).

Plasma insulin level was increased significantly in diabetic rats, while treatment with *T. chebula* aqueous extract reduced plasma insulin level significantly at 500 and 1000 mg/kg (*p* < 0.001). In addition, *T. chebula* aqueous extract treatment also improved insulin sensitivity by significantly (*p* < 0.001) decreasing HOMR-IR and increasing the insulin sensitivity index (ISI) of the treated rats (Figure 9).

### 3.7. Effect of T. chebula Treatment on Oxidative Stress Parameters

**Figure 10:**
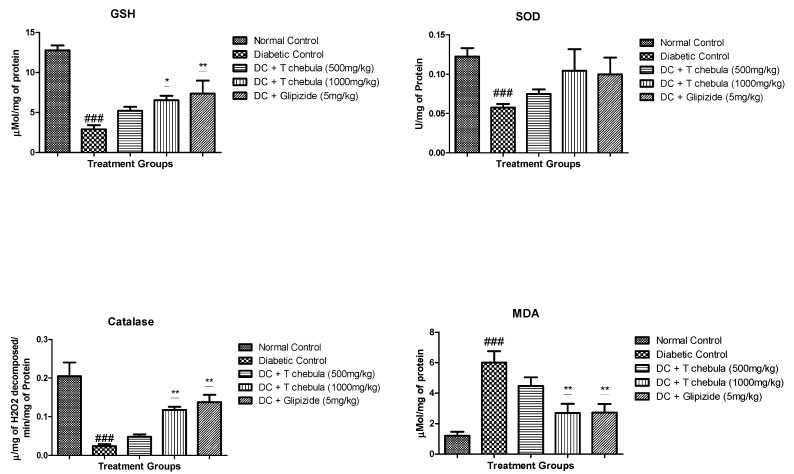
Effect of *T. chebula* aqueous extract on oxidative stress parameters. All values are expressed as Mean ± SEM (*n* = 6). * *p* < 0.05, ** *p* < 0.01 when treatment groups were compared with diabetic control. ### *p* < 0.001 when the diabetic control group was compared with the normal control group. (DC: diabetic control; *T chebula*: *Terminalia chebula*).

Diabetic rats showed a significant decrease in the levels of antioxidant enzymes such as SOD, GSH, and CAT and a substantial increase in the level of MDA in pancreatic tissue homogenate when compared to normal rats (*p* < 0.001). Treatment with a high dose of *T. chebula* aqueous extract significantly prevented the loss of GSH (*p* < 0.05) compared with diabetic control rats. *T. chebula* treatment at 1000 mg/kg significantly (*p* < 0.01) prevented the loss of CAT. Low and high dose of *T. chebula* aqueous extract treatment did not significantly prevent the loss of SOD when compared with diabetic control rats. *T. chebula* treatment at 1000 mg/kg dose and standard Glipizide treatment significantly (*p* < 0.01) prevent the formation of MDA in pancreatic tissue homogenate when compared with diabetic control rats (Figure 10).

### 3.8. Effect of T. chebula on Histopathology of Pancreatic Tissue

**Figure 11:**
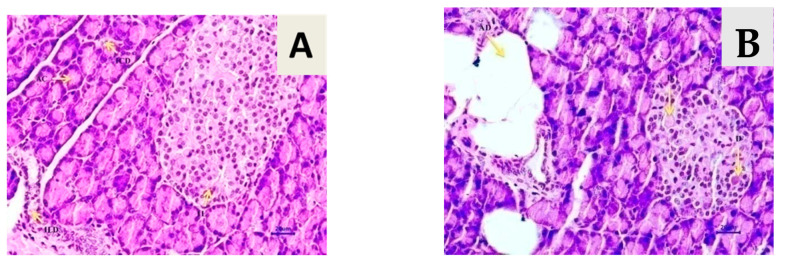

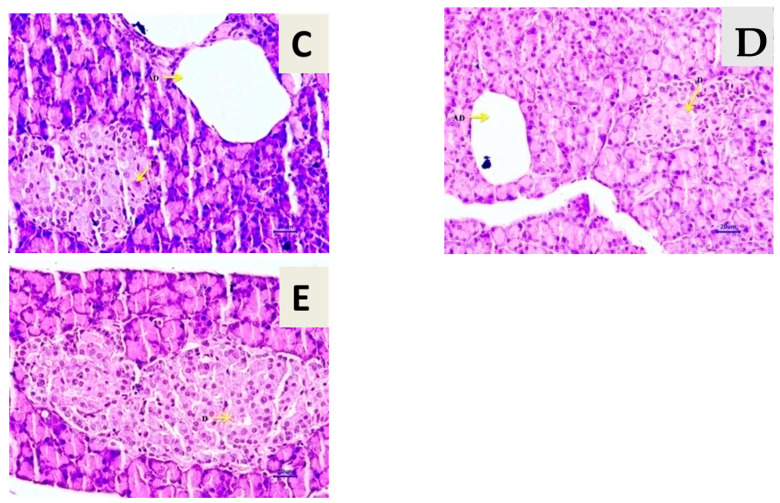
Effect of *T. chebula* on pancreatic tissue histology (H&E Stain, 400×). Histopathology of pancreatic tissue. (**A**) Normal control: pancreas showing normal histology {400×}. (**B**) Diabetes control: pancreas showing hyperplastic acini of exocrine pancreas {400×}. (**C**) Diabetic control + *T. chebula* (500 mg/kg, orally): pancreas showing hyperplastic acini of exocrine pancreas {400×}. (**D**) Diabetic + *T. chebula* (1000 mg/kg, orally): pancreas showing hyperplastic acini of exocrine pancreas, adipose tissue deposition {400×}. (**E**) Diabetic + Glipizide (5 mg/kg, orally): pancreas showing normal histology, acinus {400×}.

Microscopic examination of the pancreatic tissues of rats from the normal control group did not show any lesion of pathological significance (Figure 11). Rats from the diabetic control group and treatment groups showed various lesions in pancreatic tissue.

### 3.9. Effect of T. chebula Aqueous Extract Treatment on SIRT1 Expression

**Figure 12:**
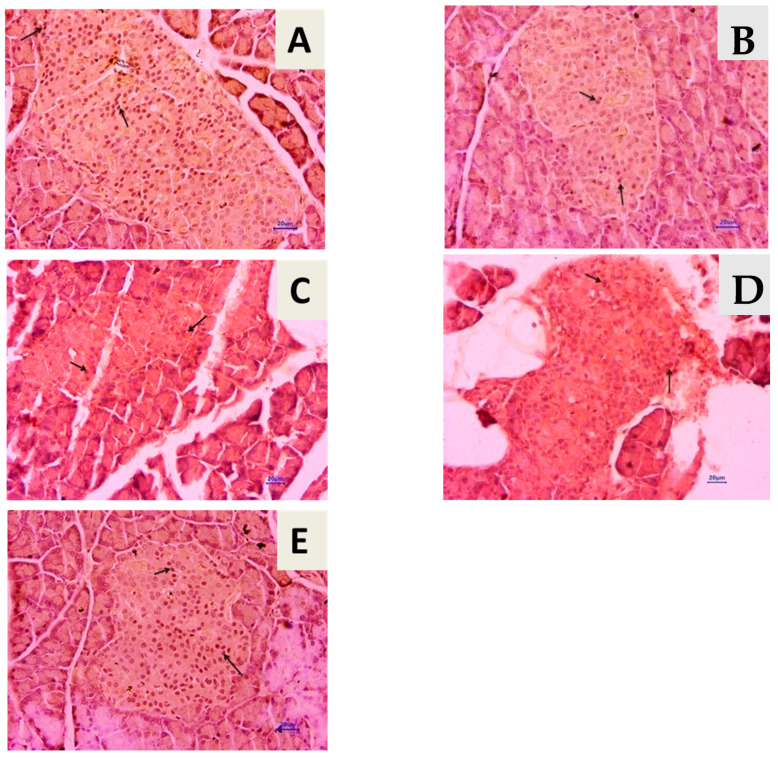
Immunohistochemical staining of pancreas (400×). Arrow showed expression of SIRT1 in the islet of Langerhans. (**A**) Normal control, (**B**) diabetes control, (**C**) diabetic + *T. chebula* (500 mg/kg, orally), (**D**) diabetic + *T. chebula* (1000 mg/kg, orally), and (**E**) diabetic + Glipizide (5 mg/kg, orally).

**Figure 13:**
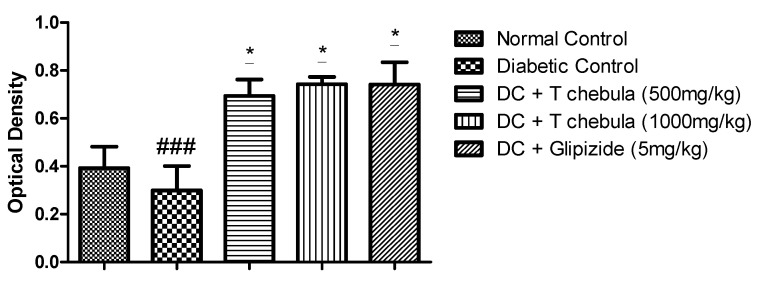
Change in optical density for expression. All values are expressed as Mean ± SEM (*n* = 3). * *p* < 0.05 when treatment groups were compared with diabetic control. ### *p* < 0.001 when the diabetic control group compared with the normal control group. (DC: diabetic control; *T chebula*: *Terminalia chebula*).

Microscopic examination of the pancreas immunohistochemically stained slides revealed SIRT1 expression, which was revealed by positive brown particles in the islet of Langerhans and acini of the exocrine pancreas (Figure 12). Untreated diabetic animals showed a reduction in the optical density of immune-stained pancreatic tissue compared to normal animals showing a decrease in SIRT1 expression in pancreatic tissue. Treatment with 500 mg/kg of aqueous extract of *T. chebula* showed an increase in the optical density of immune-stained pancreatic tissue for SIRT1 expression in pancreatic tissue, but it was not a significant increment. At the same time, 1000 mg/kg of the extract showed a significant increase in SIRT1 expression in pancreatic tissue, indicated by an increase in the optical density of immune-stained pancreatic tissue for SIRT1 (Figure 13).

## 4. Discussion

A methyl nitrosourea compound, Streptozotocin (STZ), induces diabetes in rats of type 2 diabetes mellitus [48]. The basic mechanism behind the development of diabetes due to STZ administration is specific to its alkylating chemical nature [55]. STZ is given intraperitoneally in *SD* rats, leading to pancreatic *β*-cell toxicity [49,56,57].

Researchers have established different animal models to induce type 2 diabetic conditions. In the current research study, *Sprague Dawley* rats were selected for induction of diabetes to attain pathological features comparable to type 2 diabetes mellitus in humans [58].

To induce type 2 diabetes, two important aspects the dysfunction of pancreatic *β*-cells and sensitivity of insulin reduction are important. Diet modification with a diet rich in high-fat content [59] is one of the ways to achieve a reduction in insulin sensitivity, and Streptozotocin accomplishes pancreatic *β*-cell damage. STZ in different dose levels produces different types of diabetes; a low dose of STZ is used to induce type 2 diabetes [60]. Diet modification for two weeks with a high-fat diet before administration of Streptozotocin to induce insulin resistance syndrome in rats is a well-accepted and well-studied diabetic model for developing type 2 diabetic conditions in experimental animals. [48].

In the current research work, normal rats were fed a pellet diet. In contrast, the rest all the experimental animals were given a high-fat diet for around 14 days to induce the condition of resistance to insulin. The high-fat diet should contain 40 to 60% fat composition to generate insulin resistance; thus, the high-fat diet used in this study contained around 60% fat, 25% protein, and 17% carbohydrates. Low dose, below 40mg/kg is used to induce type 2 diabetes, so in the present study, the selected dose of Streptozotocin was 35 mg/kg of body weight [61]. The induction of type 2 diabetes is characterized by hypercholesterolemia, hyperinsulinemia, and hypertriglyceridemia, conditions that are similar to human insulin resistance [62].

An alcoholic extract of *T. chebula* at 600 mg/kg showed a significant antidiabetic effect [63]. A study was performed by Kannan in type 1 diabetes in animals where alloxan was used to induce diabetes. The author mentioned that the ethanolic extract was given for 30 days, and blood glucose, plasma insulin, triglycerides, cholesterol, SGPT, SGOT, GSH, and CAT were performed and calculated. Results showed significant increase in antioxidant enzymes. This represents the effectiveness of the active phytoconstituents on type 1 diabetes [64]. Rao et al. have also reported the same type of antidiabetic effect in the chloroform extract using Streptozotocin induced diabetes. The author mentioned that the chloroform extract of *T. chebula* seeds produced a dose-dependent reduction in the blood glucose level of diabetic rats. These reports suggest the effectiveness of extracts in the treatment of diabetes mellitus. The current findings are in line with the previous studies. The current study provides first scientific evidence for the use of *T. chebula* aqueous extract in type 2 diabetes.

A significant increase in glucose level was observed in the experimental diabetic animals due to the insulin resistance by cells and damage of pancreatic *β*-cells. Treatment with *T. chebula* aqueous extract prevented pancreatic *β*-cell damage and improved insulin resistance. Results obtained from the OGTT also support this finding. The results showed that the plasma glucose level at 0, 30, 60, 90, and 120 min remained significantly higher in the diabetic control group when compared with the normal control group (*p* < 0.001) and other treatment groups (*p* < 0.05 and 0.01). *T. chebula* aqueous extract treatment also decreased plasma insulin levels significantly in diabetic animals.

In addition, extract treatment helped increase insulin sensitivity and ISI and decrease HOMA-IR at both the selected doses, indicating improvement in insulin utilization and glucose metabolism. Insulin resistance is also linked with dyslipidemia in type 2 diabetes mellitus. Alteration in lipoprotein metabolism containing triglyceride and a rise in hepatic lipase activity results in elevated levels of LDL, cholesterol, and triglyceride with a reduction in HDL levels in diabetics [65,66,67]. The plasma lipid profile was significantly elevated in the diabetic control group. Treatment with *T. chebula* aqueous extract improved lipid profile significantly. Amplified activities of enzymes in the liver, such as ALT and AST, are the marker enzymes for injury in hepatic cells. Augmentation in the activity of these enzymes results in metabolic syndrome, type 2 diabetes, and insulin resistance [68,69]. *T. chebula* aqueous extract treatment controlled hyperglycemic conditions and prevented liver tissue from being damaged. As a result, a considerable reduction in the AST and ALT was observed in the *T. chebula* treatment group compared to diabetic rats. In type 2 diabetes, protein glycation is a natural process, including the formation of Glycohemoglobin, where glucose is reacted with hemoglobin at the N-terminal end of the *β*-chain, which is responsible for the formation of Glycohemoglobin [70]. HbA1c does not give an idea of daily blood glucose; it indicates an average of blood glucose over the past few days [71]. It is well known that the normal limit for glycohemoglobin is 3 to 6%; however, type 2 diabetes increases up to 6 to 12% due to persistent hyperglycemia [69]. In the present study, *T. chebula* treatment controlled blood glucose levels and thus prevented the formation of Glycohemoglobin when compared with diabetic rats.

Oxidative damage to the pancreatic tissue due to prolonged hyperglycemia will have side effects on the concentration of antioxidant enzymes in the pancreas. Along with CAT and SOD, GSH looks after the pancreas from H_2_O_2_ damage, alkoxy radicals, and superoxide [72,73,74,75]. Tiwari et al. (2013) mentioned that decreased GSH levels might be one of the factors in oxidative damage in type 2 diabetes [73]. The *T. chebula* treatment prevented pancreatic tissue from oxidative damage by increasing the concentration of GSH, CAT, and SOD and prohibiting the production of reactive aldehyde in tissue via its antioxidant mechanism. Histopathological examination of the pancreatic tissue revealed the presence of inflammatory, degenerative, necrotic, and hyperplastic lesions in the endocrine and exocrine parts of the pancreas in diabetic animals. Atrophy of islets of Langerhans was also observed in diabetic control animals. Results also showed the presence of adipose deposition at the acini lobule, degeneration of the endocrine pancreas, and lymphocytic infiltration. Treatment with *T. chebula* aqueous extract showed decrease in pancreatic damage caused due to diabetic condition. Certain evidence suggests that SIRT1 has a protective role in diabetic condition.

SIRT1, a NAD-dependent class III histone deacetylase that regulates glucose homeostasis by regulating hepatic glucose production, lipid metabolism, insulin production, and sensitivity [76,77]. SIRT1 is a protein family member with a broad and vital role in regulating cellular health. Some studies have reported that up-regulated expression of SIRT1 hinders the formation and development of pancreatic tissue fibrosis in type 2 diabetes in rats. In addition, SIRT1 controls glucose homeostasis by regulating insulin production, lipid metabolism, insulin sensitivity, and hepatic glucose production [54]. In the current research study, considerably reduced levels of SIRT1 in pancreatic tissue were observed in diabetic rats. However, *T. chebula* aqueous extracts improve the SIRT1 expression indicating protection against pancreatic tissue damage.

## 5. Conclusions

The aqueous extract of *Terminalia chebula* decreased oxidative stress and increased the expression of SIRT1 in diabetic rats. This study’s results indicate that *T. chebula* aqueous extract has a significant antidiabetic effect.

## Figures and Tables

**Table 1:** Chromatographic data of ellagic acid and gallic acid standards and extract contents.

Name of Marker	Concentration µg/mL	Peak Area	Volume of Solution µL	Concentration
Ellagic acid	100	0.01724	8	0.0018
Gallic acid	100	0.00498	8	0.0077

## Data Availability

The original contributions presented in this study are included in the article; further inquiries can be directed to the corresponding authors.

## Abbreviations

STZ	Streptozotocin;
ISI	Insulin sensitivity index;
HOMA-IR	Homeostatic Model Assessment of Insulin Resistance;
SIRT1	Silent Mating Type Information Regulation 2 Homolog;
SD rats	*Sprague Dawley* rats;
TC	Total cholesterol;
TG	Triglyceride;
HDL	High-density lipoprotein;
LDL	Low-density lipoprotein;
AST	Aspartate Aminotransferase;
ALT	Alanine Aminotransferase;
H&E	Hematoxylin and Eosin;
IHC	Immunohistochemistry;
ANOVA	Analysis of variance;
NFBG	Non-Fasting Blood Glucose;
Na CMC	Sodium Carboxy Methyl Cellulose;
HPTLC	High-Performance Thin Layer Chromatography.

## References

[1] Lecka-Czernik B. (2017). Diabetes, bone and glucose-lowering agents: Basic biology. Diabetologia.

[2] (2019). International Diabetes Federation IDF Diabetes Atlas 9th edition south-east Asia. Int. Diabetes Fed..

[3] International Diabetes Federation (2021). IDF Diabetes Atlas.

[4] International Diabetes Federation (2017). IDF Diabetes Atlas.

[5] Hu F.B., Manson J.E., Stampfer M.J., Colditz G., Liu S., Solomon C.G., Willett W.C. (2001). Diet, lifestyle, and the risk of type 2 diabetes mellitus in women. N. Engl. J. Med..

[6] Kiani R. (2022). Dyslipidemia. Practical Cardiology.

[7] Jialal I., Singh G. (2019). Management of diabetic dyslipidemia: An update. World J. Diabetes.

[8] Berberich A.J., Hegele R.A. (2022). A Modern Approach to Dyslipidemia. Endocr. Rev..

[9] Subramanian S., Chait A. (2000). Dyslipidemia in Diabetes. Encyclopedia of Endocrine Diseases.

[10] Olokoba A.B., Obateru O.A., Olokoba L.B. (2012). Type 2 diabetes mellitus: A review of current trends. Oman Med. J..

[11] Freeman J.S. (2010). The increasing epidemiology of diabetes and review of current treatment algorithms. J. Am. Osteopat. Assoc..

[12] Eidi A., Eidi M., Esmaeili E. (2006). Antidiabetic effect of garlic (*Allium sativum* L.) in normal and streptozotocin-induced diabetic rats. Phytomedicine.

[13] Saikat A.S.M., Hossain R., Mina F.B., Das S., Khan I.N., Mubarak M.S., Islam M.T. (2022). Antidiabetic Effect of Garlic. Rev. Bras. Farmacogn..

[14] Jana K., Bera T.K., Ghosh D. (2015). Antidiabetic effects of *Eugenia jambolana* in the streptozotocin-induced diabetic male albino rat. Biomark. Genom. Med..

[15] Joseph B., Jini D. (2013). Antidiabetic effects of *Momordica charantia* (bitter melon) and its medicinal potency. Asian Pac. J. Trop. Dis..

[16] Sethi J., Sood S., Seth S., Talwar A. (2004). Evaluation of hypoglycemic and antioxidant effect of *Ocimum sanctum*. Indian J. Clin. Biochem..

[17] Suanarunsawat T., Anantasomboon G., Piewbang C. (2016). Anti-diabetic and anti-oxidative activity of fixed oil extracted from *Ocimum sanctum* L. leaves in diabetic rats. Exp. Ther. Med..

[18] Adeneye A.A., Amole O.O., Adeneye A.K. (2006). Hypoglycemic and hypocholesterolemic activities of the aqueous leaf and seed extract of *Phyllanthus amarus* in mice. Fitoterapia.

[19] Raphael K.R., Sabu M.C., Kuttan R. (2002). Hypoglycemic effect of methanol extract of *Phyllanthus amarus* Schum & Thonn on alloxan induced diabetes mellitus in rats and its relation with antioxidant potential. Indian J. Exp. Biol..

[20] Lawson-Evi P., Eklu-Gadeg K., Agbonon A., Aklikokou K., Creppy E., Gbeassor M. (2011). Antidiabetic Activity of *Phyllanthus amarus* Schum and Thonn (Euphorbiaceae) on Alloxan Induced Diabetes in Male Wistar Rats. J. Appl. Sci..

[21] Dhanabal S.P., Kokate C.K., Ramanathan M., Kumar E.P., Suresh B. (2006). Hypoglycaemic activity of *Pterocarpus marsupium* Roxb. Phyther. Res..

[22] Joladarashi D., Chilkunda N.D., Salimath P.V. (2014). Glucose uptake-stimulatory activity of *Tinospora cordifolia* stem extracts in Ehrlich ascites tumor cell model system. J. Food Sci. Technol..

[23] Geberemeskel G.A., Debebe Y.G., Nguse N.A. (2019). Antidiabetic Effect of Fenugreek Seed Powder Solution (*Trigonella foenum-graecum* L.) on Hyperlipidemia in Diabetic Patients. J. Diabetes Res..

[24] Gorelick J., Rosenberg R., Smotrich A., Hanuš L., Bernstein N. (2015). Hypoglycemic activity of withanolides and elicitated *Withania somnifera*. Phytochemistry.

[25] Ali Z., Khan I. (2009). Chemical Constituents of Terminalia chebula. Planta Med..

[26] Sotoudeh R., Hadjzadeh M.-A.-R., Gholamnezhad Z., Aghaei A. (2019). The anti-diabetic and antioxidant effects of a combination of *Commiphora mukul*, *Commiphora myrrha* and *Terminalia chebula* in diabetic rats. Avicenna J. Phytomed..

[27] Kumar G.P.S., Arulselvan P., Kumar D.S., Subramanian S.P. (2006). Anti-diabetic activity of fruits of *Terminalia chebula* on streptozotocin induced diabetic rats. J. Health Sci..

[28] Sasidharan I., Sundaresan A., Nisha V.M., Kirishna M.S., Raghu K.G., Jayamurthy P. (2012). Inhibitory effect of *Terminalia chebula* Retz. fruit extracts on digestive enzyme related to diabetes and oxidative stress. J. Enzyme Inhib. Med. Chem..

[29] Moradi B., Abbaszadeh S., Shahsavari S., Alizadeh M., Beyranvand F. (2018). The most useful medicinal herbs to treat diabetes. Biomed. Res. Ther..

[30] Kim H.G., Lee J.S., Lee J.S., Han J.M., Son C.G. (2012). Hepatoprotective and antioxidant effects of Myelophil on restraint stress-induced liver injury in BALB/c mice. J. Ethnopharmacol..

[31] Choi M.K., Kim H.G., Han J.M., Lee J.S., Lee J.S., Chung S.H., Son C.G. (2015). Hepatoprotective Effect of *Terminalia chebula* against t-BHP-Induced Acute Liver Injury in C57/BL6 Mice. Evid. Based Complement. Altern. Med..

[32] Pfundstein B., El Desouky S.K., Hull W.E., Haubner R., Erben G., Owen R.W. (2010). Polyphenolic compounds in the fruits of Egyptian medicinal plants (*Terminalia bellerica*, *Terminalia chebula* and *Terminalia horrida*): Characterization, quantitation and determination of antioxidant capacities. Phytochemistry.

[33] Yakaew S., Itsarasook K., Ngoenkam J., Jessadayannamaetha A., Viyoch J., Ungsurungsie M. (2016). Ethanol extract of *Terminalia chebula* fruit protects against UVB-induced skin damage. Pharm. Biol..

[34] Lee H.S., Won N.H., Kim K.H., Lee H., Jun W., Lee K.W. (2005). Antioxidant effects of aqueous extract of *Terminalia chebula* in Vivo and in Vitro. Biol. Pharm. Bull..

[35] Naik G.H., Priyadarsini K.I., Naik D.B., Gangabhagirathi R., Mohan H. (2004). Studies on the aqueous extract of *Terminalia chebula* as a potent antioxidant and a probable radioprotector. Phytomedicine.

[36] Saha S., Verma R.J. (2016). Antioxidant activity of polyphenolic extract of *Terminalia chebula* Retzius fruits. J. Taibah Univ. Sci..

[37] Chang C.L., Lin C.S. (2012). Phytochemical composition, antioxidant activity, and neuroprotective effect of *Terminalia chebula* Retzius extracts. Evid. Based Complement. Altern. Med..

[38] Cheng H.Y., Lin T.C., Yu K.H., Yang C.M., Lin C.C. (2003). Antioxidant and free radical scavenging activities of *Terminalia chebula*. Biol. Pharm. Bull..

[39] Sheng Z., Zhao J., Muhammad I., Zhang Y. (2018). Optimization of total phenolic content from *Terminalia chebula* Retz. fruits using response surface methodology and evaluation of their antioxidant activities. PLoS ONE.

[40] Li K., Diao Y., Zhang H., Wang S., Zhang Z., Yu B., Huang S., Yang H. (2011). Tannin extracts from immature fruits of *Terminalia chebula* Fructus Retz. promote cutaneous wound healing in rats. BMC Complement. Altern. Med..

[41] Suguna L., Singh S., Sivakumar P., Sampath P., Chandrakasan G. (2002). Influence of *Terminalia chebula* on dermal wound healing in rats. Phyther. Res..

[42] Ali S.K., Hamed A.R., Soltan M.M., Hegazy U.M., Elgorashi E.E., El-Garf I.A., Hussein A.A. (2013). In-vitro evaluation of selected Egyptian traditional herbal medicines for treatment of Alzheimer disease. BMC Complement. Altern. Med..

[43] Kalra P., Karwasra R., Gupta Y.K., Ray S.B., Singh S. (2019). *Terminalia chebula* supplementation attenuates cisplatin-induced nephrotoxicity in Wistar rats through modulation of apoptotic pathway. Nat. Prod. Res..

[44] Ding G., Lu Y.R., Ji C.R., Liu Y.Z. (2001). Analysis of tannins in Fructus Chebulae and its confusion varieties by HPCE. Yaoxue Xuebao.

[45] Juang L.-J., Sheu S.-J., Lin T.-C. (2004). Determination of hydrolyzable tannins in the fruit of *Terminalia chebula* Retz. by high-performance liquid chromatography and capillary electrophoresis. J. Sep. Sci..

[46] Kundu A.P., Mahato S.B. (1993). Triterpenoids and their glycosides from *Terminalia chebula*. Phytochemistry.

[47] Kulkarni Y.A., Panjabi R., Patel V., Tawade A., Gokhale A. (2013). Effect of *Gmelina arborea* Roxb in experimentally induced infl ammation and nociception. J. Ayurveda Integr. Med..

[48] Srinivasan K., Viswanad B., Asrat L., Kaul C.L., Ramarao P. (2005). Combination of high-fat diet-fed and low-dose streptozotocin-treated rat: A model for type 2 diabetes and pharmacological screening. Pharmacol. Res..

[49] Kulkarni Y.A., Garud M.S. (2016). *Bauhinia variegata* (Caesalpiniaceae) leaf extract: An effective treatment option in type I and type II diabetes. Biomed. Pharmacother..

[50] Ellman G.L. (1959). Tissue sulfhydryl groups. Arch. Biochem. Biophys..

[51] Ohkawa H., Ohishi N., Yagi K. (1979). Assay for lipid peroxides in animal tissues by thiobarbituric acid reaction. Anal. Biochem..

[52] Paoletti F., Mocali A., Aldinucci D. (1990). Superoxide-driven NAD(P)H oxidation induced by EDTA-manganese complex and mercaptoethanol. Chem. Biol. Interact..

[53] Garud M.S., Kulkarni Y.A. (2017). Eugenol ameliorates renal damage in streptozotocin-induced diabetic rats. Flavour Fragr. J..

[54] Oza M.J., Kulkarni Y.A. (2018). Formononetin treatment in type 2 diabetic rats reduces insulin resistance and hyperglycemia. Front. Pharmacol..

[55] Lenzen S. (2008). The mechanisms of alloxan- and streptozotocin-induced diabetes. Diabetologia.

[56] Murata M., Takahashi A., Saito I., Kawanishi S. (1999). Site-specific DNA methylation and apoptosis: Induction by diabetogenic streptozotocin. Biochem. Pharmacol..

[57] LeDoux S.P., Woodley S.E., Patton N.J., Wilson G.L. (1986). Mechanisms of nitrosourea-induced β-cell damage: Alterations in DNA. Diabetes.

[58] Båvenholm P.N., Pigon J., Östenson C.G., Efendic S. (2001). Insulin sensitivity of suppression of endogenous glucose production is the single most important determinant of glucose tolerance. Diabetes.

[59] Ohtsubo K., Chen M.Z., Olefsky J.M., Marth J.D. (2011). Pathway to diabetes through attenuation of pancreatic beta cell glycosylation and glucose transport. Nat. Med..

[60] Gheibi S., Kashfi K., Ghasemi A. (2017). A practical guide for induction of type-2 diabetes in rat: Incorporating a high-fat diet and streptozotocin. Biomed. Pharmacother..

[61] Zhang F.L., Ye C., Li G., Ding W., Zhou W., Zhu H., Chen G., Luo T., Guang M., Liu Y. (2003). The rat model of type 2 diabetic mellitus and its glycometabolism characters. Exp. Anim..

[62] Reaven G.M. (1991). Insulin resistance, hyperinsulinemia, hypertriglyceridemia, and hypertension: Parallels between human disease and rodent models. Diabetes Care.

[63] Eltimamy M., Elshamarka M., Aboelsaad M., Sayed M., Moawad H. (2022). Effects of alcoholic extract of *Terminalia chebula* dried fruit on blood biochemical profile in diabetic rats. J. Diabetes Metab. Disord..

[64] Kannan V.R., Rajasekar G.S., Rajesh P., Balasubram V., Ramesh N., Solomon E.K., Nivas D., Chandru S. (2012). Anti-diabetic Activity on Ethanolic Extracts of Fruits of *Terminalia chebula* Retz. Alloxan Induced Diabetic Rats. Am. J. Drug Discov. Dev..

[65] Hirano T. (2018). Pathophysiology of diabetic dyslipidemia. J. Atheroscler. Thromb..

[66] Shanik M.H., Xu Y., Skrha J., Dankner R., Zick Y., Roth J. (2008). Insulin resistance and hyperinsulinemia: Is hyperinsulinemia the cart or the horse?. Diabetes Care.

[67] Krauss R.M. (2004). Lipids and lipoproteins in patients with type 2 diabetes. Diabetes Care.

[68] Mohamed J., Nazratun Nafizah A.H., Zariyantey A.H., Budin S.B. (2016). Mechanisms of diabetes-induced liver damage: The role of oxidative stress and inflammation. Sultan Qaboos Univ. Med. J..

[69] Shen J., Zhang J., Wen J., Ming Q., Zhang J., Xu Y. (2015). Correlation of serum alanine aminotransferase and aspartate aminotransferase with coronary heart disease. Int. J. Clin. Exp. Med..

[70] Sherwani S.I., Khan H.A., Ekhzaimy A., Masood A., Sakharkar M.K. (2016). Significance of HbA1c test in diagnosis and prognosis of diabetic patients. Biomark. Insights.

[71] Makris K., Spanou L. (2011). Is there a relationship between mean blood glucose and glycated hemoglobin?. J. Diabetes Sci. Technol..

[72] Tsai K., Wang S.S., Chen T.S., Kong C.W., Chang F.Y., Lee S.D., Lu F.J. (1998). Oxidative stress: An important phenomenon with pathogenetic significance in the progression of acute pancreatitis. Gut.

[73] Tiwari B.K., Pandey K.B., Abidi A.B., Rizvi S.I. (2013). Markers of Oxidative Stress during Diabetes Mellitus. J. Biomark..

[74] Schoenberg M.H., Büchler M., Baczako K., Bültmann B., Younes M., Gasper M., Kirchmayr R., Beger H.G. (1991). The involvement of oxygen radicals in acute pancreatitis. Klin. Wochenschr..

[75] Pasupuleti V.R., Arigela C.S., Gan S.H., Salam S.K.N., Krishnan K.T., Rahman N.A., Jeffree M.S. (2020). A review on oxidative stress, diabetic complications, and the roles of honey polyphenols. Oxidative Med. Cell. Longev..

[76] Shoba B., Lwin Z.M., Ling L.S., Bay B.H., Yip G.W., Kumar S.D. (2009). Function of sirtuins in biological tissues. Anat. Rec..

[77] Pulla V.K., Battu M.B., Alvala M., Sriram D., Yogeeswari P. (2012). Can targeting SIRT-1 to treat type 2 diabetes be a good strategy? A review. Expert Opin. Ther. Targets.

